# Diagnostic Performance of Plasma Phospho‐tau217 for Alzheimer's Disease in a Large Memory Clinic Cohort

**DOI:** 10.1002/alz70856_106784

**Published:** 2026-01-08

**Authors:** Anuradha Sehrawat, Xuemei Zeng, Rebecca A Deek, Michel N Nafash, Jeremy M. Gu, Lamia Choity, Tara K Lafferty, Marissa F Farinas, Rocco B Mercurio, Cristy Matan, Alexandra Gogola, Julia K. Kofler, Dana L Tudorascu, C. Elizabeth Shaaban, Jennifer H Lingler, Tharick A Pascoal, William E Klunk, Victor L. Villemagne, Sarah B Berman, Robert Sweet, Milos D. Ikonomovic, Beth E. Snitz, Ann D Cohen, M. Ilyas Kamboh, Oscar L Lopez, Thomas K Karikari

**Affiliations:** ^1^ University of Pittsburgh, Pittsburgh, PA, USA; ^2^ University of Pittsburgh School of Medicine, Pittsburgh, PA, USA; ^3^ University of Pittsburgh Alzheimer's Disease Research Center (ADRC), Pittsburgh, PA, USA; ^4^ University of Pittsburgh, School of Medicine, Pittsburgh, PA, USA; ^5^ UPMC, Pittsburgh, PA, USA; ^6^ VA Pittsburgh Healthcare System, Pittsburgh, PA, USA

## Abstract

**Background:**

Phospho‐tau217 (*p*‐tau217) has emerged as a leading blood‐based biomarker for Alzheimer's disease (AD) and has been recommended by an Alzheimer's Association Workgroup as a biomarker for AD pathology. However, its translational performance in clinical settings remains to be explored. The objective of this study was to evaluate the concordance between *p*‐tau217 predicted AD pathology and the clinical diagnosis of AD in a large memory clinic cohort to address this question.

**Methods:**

Participants at the University of Pittsburgh Alzheimer's Disease Research Center (Pitt‐ADRC) provided blood samples and underwent ADRC classification scheme‐based clinical diagnosis. *APOE* genotyping was conducted using TaqMan assays. Plasma *p*‐tau217 levels were quantified using the Quanterix ALZpath Single Molecule Array (Simoa) assay. A sub‐cohort with amyloid PET data was used to establish the *p*‐tau217 cutpoints. Both a single‐cutpoint approach based on the Youden index and a two‐cutpoint approach, with low and high cutpoints determined by 99% sensitivity and 95% specificity, respectively, were used to evaluate the agreement between *p*‐tau217 and clinical diagnosis status. Logistic regression was used to statistically test the interaction between *p*‐tau217 and demographic and genetic covariates.

**Results:**

This study included 3,788 participants (59.6% female; 47.8% *APOE ε4* carriers), of whom 2,014 had a clinical diagnosis of AD. Using a single‐cutoff approach with a threshold of 0.547 pg/mL, *p*‐tau217 demonstrated 84.3% sensitivity, 76.0% specificity, and an overall accuracy of 82.0% in interpreting AD status. The two‐cutpoint approach, with low and high cutpoints of 0.321 and 0.935, improved the diagnostic accuracy to 88.0%, with sensitivity and specificity of 89.9% and 91.7%, respectively, and 1360 (35.9%) in the intermediate zone. With the single‐cutpoint approach, individuals with high *p*‐tau217 were associated with an odds ratio of 17.1 of having AD compared to those with low *p*‐tau217. Sex, but not race, and *APOE ε4* carrier status significantly moderated the association between *p*‐tau217 status and clinical diagnosis, with women having an odds ratio of 20.9 compared to men of 12.1 (*p* = 0.010).

**Conclusion:**

Our results demonstrated that using *p*‐tau217 as a surrogate biomarker for AD pathology shows strong concordance with clinical diagnosis and can be a promising tool for enhancing clinical decision‐making.

